# Transdiagnostic Anxiety-Related Increases in Information Sampling are Associated With Altered Valuation

**DOI:** 10.5334/cpsy.100

**Published:** 2024-11-06

**Authors:** Amy M. Rapp, Brandon K. Ashinoff, Seth Baker, H. Blair Simpson, Guillermo Horga

**Affiliations:** 1 Department of Psychiatry, Columbia University, US; 2 New York State Psychiatric Institute, US; 3 Department of Psychiatry, Icahn School of Medicine at Mount Sinai, US; 4 University at Buffalo, Jacobs School of Medicine and Biomedical Sciences, US

**Keywords:** Information sampling, beads task, trait anxiety, valuation, theory-driven computational modeling, transdiagnostic

## Abstract

Excessive information sampling in psychiatric patients characterized by high trait anxiety has been inconsistently linked with alterations in inferential and valuation processes. Methodological limitations could account in part for these inconsistencies. To address this, computational models of inference and valuation were applied to data collected from a transdiagnostic sample of adults with and without an anxiety or compulsive disorder using a version of the beads task with enhanced experimental controls. Participants diagnosed with an anxiety or compulsive disorder (*n* = 35) and healthy controls (*n* = 23) completed the beads task with three majority-to-minority ratios of blue versus green beads (60:40, 75:25, 90:10). First, a Bayesian belief-updating model was fit to quantify the iterative process by which new information (bead color) and prior beliefs were integrated to influence current beliefs about jar identity. Next, a parameterized partially observable Markov decision process model was used to parse the contribution of value-based decisions to sampling behavior and included a relative subjective cost parameter, *C_sub_*, for each bead-ratio condition. Higher trait anxiety was associated with more draws-to-decision, most robustly in the 90:10 bead-ratio condition. Only relative subjective cost of sampling decisions, and not inferential differences in weighting of new or old information, satisfactorily accounted for this relation. Specifically, lower *C_sub_(0.9)* was associated with more trait anxiety and more draws-to-decision. In a condition with high objective evidence strength, transdiagnostic trait-anxiety-related increases in information sampling were explained by a cost-benefit analysis where relatively higher subjective cost was assigned to an incorrect guess, highlighting valuation as a potential treatment target for future research.

The ability to gather an appropriate amount of information about one’s environment and use this information effectively is critical for adaptive decision-making. In patients with anxiety and compulsive disorders, such as obsessive-compulsive disorder (OCD) and generalized anxiety disorder (GAD), these processes may go awry, manifesting in a range of clinical symptoms. For example, individuals with OCD commonly engage in compulsive checking (Ruscio et al., 2010; Strauss et al., 2020) in response to an exaggerated sense of responsibility for preventing an anticipated, seriously harmful event (Rachman, 2002) (e.g., repeatedly checking that the stove has been turned off to avoid starting a fire), and those with GAD often solicit excessive reassurance from others (Woody & Rachman, 1994) (e.g., repeatedly contacting a romantic partner to confirm that their relationship is secure).

This clinical expression of excessive information sampling could be driven by distinct mechanisms, in particular, deficits in inferential ability and value-based decision-making. When inferential processes are altered, individuals may demonstrate difficulty with implementing logical reasoning to draw a conclusion based on the information provided. In individuals with schizophrenia, for example, increased information sampling was explained by this kind of deficit, specifically greater weighting of information presented earlier in the inferential process (Baker et al., 2019). In contrast, when there are alterations in valuation, as has been seen among individuals diagnosed with anxiety and compulsive disorders (Maia et al., 2008; Menzies et al., 2008), greater information sampling might be attributable to a lower perceived value of additional sampling or greater subjective cost of making an incorrect choice. Given that there are multiple pathways that could give rise to the same apparent behavior, disentangling these underlying processes would provide more precise understanding of the neurocognitive mechanisms of inappropriate information sampling and inform potential treatment targets.

Eliciting information sampling behaviors in humans has been accomplished by using a range of decision-making experimental tasks in laboratory studies. One of the most used is the beads task (Huq et al., 1988), in which participants are asked to guess the color of the majority of beads contained in a hidden jar. The number of beads drawn prior to making a choice about the identity of the jar (i.e., draws-to-decision) is generally used as the main outcome and considered a behavioral measure of information sampling. Different versions of the beads task have been used in clinical samples, and studies including samples of individuals diagnosed with anxiety and compulsive disorders have produced mixed results. For example, relative to healthy controls, individuals with OCD have been shown to request more (Pélissier & O’Connor, 2002; Volans, 1976; Voon et al., 2017), fewer (Grassi et al., 2015; for review, So et al., 2016), and the same number of beads (Fear & Healy, 1997; Hezel et al., 2019; Jacobsen et al., 2012; Morein-Zamir et al., 2020) before making a decision.

Several methodological limitations might account for such discrepant findings. These include the use of information sampling tasks that lack appropriate controls for decision-making processes or task design elements (e.g., time-pressure to make a decision, low motivation; for review, Ashinoff et al., 2021), data-analytic methods unable to parse the algorithmic mechanisms underlying altered information sampling, and reliance on categorical diagnostic group comparisons. To address this, researchers have increasingly called for the use of well-controlled experimental tasks that reflect underlying neurobiological processes and data analysis using theoretical approaches (Ferrante & Gordon, 2021; Huys et al., 2016). Research designed in this way has elucidated inferential and valuation processes as mechanisms accounting for greater information gathering in some clinical samples. For example, data analysis with computational models showed that adolescents with OCD demonstrated higher decision thresholds on a sequential information gathering task that were driven by alterations in subjective costs of information sampling (Hauser et al., 2017).

Few studies have examined both inference and valuation together in relation to dimensional clinical constructs (rather than diagnostic group comparisons) in transdiagnostic samples. Indeed, most studies of anxiety and information sampling have been conducted in general, non-clinical samples. Thus, the goal of this study was to examine the association of information sampling and a dimensional measure of trait anxiety, a clinical characteristic common to compulsive and anxiety disorders, in a transdiagnostic sample that included adults diagnosed with anxiety and compulsive disorders. To more precisely clarify the mechanisms of variability in this association, we used an adapted version of the beads task (Baker et al., 2019) that improved upon previous designs by accounting for potential confounds and applied computational models of inference and value-based decision-making to these data. We expected that trait anxiety would be transdiagnostically associated with greater information sampling (i.e., more draws-to-decision) and aimed to disentangle the distinct and/or interacting contributions of inferential alterations in information weighting and value-based sampling decisions to this association.

## Methods

### Participants and Procedures

Sixty-two participants were recruited as part of a study approved by the New York State Psychiatric Institute Institutional Review Board. Participants who met diagnostic criteria for OCD, GAD, social anxiety disorder (SAD), panic disorder, agoraphobia, specific phobia, illness anxiety disorder, or adult separation anxiety disorder and had not been taking psychotropic medication or receiving cognitive-behavioral therapy for at least four weeks were included in the study. Psychiatric diagnoses were determined by MA- and Ph.D.-level assessors using the Structured Clinical Interview for DSM-5. Healthy controls had no current or lifetime DSM-5 diagnoses. Exclusion criteria for all participants included current comorbid diagnosis of major depressive disorder, psychotic disorder, bipolar disorder, alcohol and/or substance use disorder, acute suicidal risk, and any major medical condition. Participants provided written informed consent and completed a battery of self-report questionnaires and neurocognitive tasks (relevant measures described below).

#### Clinical Measure

The State-Trait Anxiety Inventory (STAI; Spielberger, 1983) is a 40-item self-report questionnaire that produces measures of state (e.g., “I am tense”) and trait (e.g., “I worry too much over something that really doesn’t matter”) anxiety. Items are rated on a Likert scale from 1 (“Almost never”) to 4 (“Almost always”). Scores for each subscale range from 20–80 with a score of 40 considered indicative of clinically elevated state or trait anxiety. Internal consistency of the STAI has been found to be good to excellent in other samples (*α* = 0.86–0.95; Spielberger, 1983).

#### Beads Task

This study used a modified version of the beads task (Baker et al., 2019) that built on previous designs (Furl & Averbeck, 2011; Huq et al., 1988) by accounting for decision-making processes or task design elements that could influence task performance (e.g., working memory deficits, miscomprehension of instructions; for review, Ashinoff et al., 2021).

Participants were provided detailed instructions including several practice trials and a comprehension quiz. Participants were told that at the start of each trial, either a jar with mostly blue beads or a jar with mostly green beads was selected at random and concealed from them and their goal was to correctly guess the jar’s identity, aided by drawing up to a maximum of eight beads from the hidden jar. Each jar had a majority-to-minority ratio of green or blue beads (“bead-ratio”) that was shown to the participant before the start of the trial. These ratios could be 60:40, 75:25, 90:10, or 100:0. Before the start of the task, participants were shown a $30 cash sum which was placed in a metal box next to the participant throughout the experimental session. Participants were informed that this sum would be available for each trial, with $0.30 deducted for each bead drawn from the hidden jar and $15 deducted for an incorrect guess of the jar’s identity. To increase engagement and mitigate sequential effects, participants were told that at the end of the task, the amount of money retained on one trial would be randomly selected to be paid out. This encouraged participants to perform consistently throughout. There was a 30-minute minimum task duration to discourage participants from hastily completing the task. Participants completed 70 trials total which included 20 trials with 60:40, 75:25, and 90:10 bead-ratios, and 10 “catch” condition trials with the 100:0 bead-ratio. Trials were presented in a pseudo-random order. A full description of the sequences used is available in the supplement of Baker et al. (2019).

Each trial began with the presentation of a fixation cross. Participants were then asked to provide an estimate of the probability of the hidden jar’s identity before drawing any beads which was indicated on a visual analogue scale ranging from 0% to 100%. This provided a baseline measure of “prior beliefs.” Next, participants were given the choice of drawing a bead or guessing the jar’s identity. The participant’s current winnings, the sequence of beads already selected, and the bead-ratio condition were also displayed. Participants could complete trials in a self-paced manner without time constraints to prevent them from feeling rushed, which has been linked to decreased draws-to-decision (for review, Ashinoff et al., 2021).

### Data Analysis

Analyses included only those individuals who identified the “correct” jar at the end of the beads sequence with greater than 62% accuracy. An accuracy cut-off was determined via a binomial test (at *p* = 0.05), which gives the number of correct trials (out of the 70 trials) that would exceed a 50% chance of success (42 correct trials out of 70, or 60%). Participants were also excluded based on task miscomprehension criteria established *a priori* (Baker et al., 2019). Specifically, participants were excluded if they did not demonstrate: (1) a significant main effect of bead-ratio condition on draws-to-decision behavior in a one-way ANOVA, indicating adjustment in the number of beads requested as evidence strength increased in each condition, (2) selection of fewer beads in the 100:0 bead-ratio condition than the 60:40 bead-ratio condition (i.e., significant least significant difference test), and (3) an average of fewer than three draws-to-decision in the 100:0 bead-ratio condition. Participants who demonstrated outlier model parameter estimates, defined as a value greater than three scaled median absolute deviation (MAD) away from the median (Leys et al., 2013), were also excluded. Post-hoc tests were conducted to examine robustness of results to stricter exclusion criteria.

#### Belief-Updating Model

Following previous work (Furl & Averbeck, 2011), changes in probability estimates were modelled on each draw using methods described in detail by Baker and colleagues (2019). Probability estimates were presumed to more directly approximate belief-updating compared to draws-to-decision, given previous research demonstrating that draws-to-decision behavior also depends on other cognitive processes or task-design elements unrelated to inference. Informed by Bayes’ theorem, this model quantified the iterative process by which a new sample of information (i.e., bead color of a newly drawn bead) and prior beliefs are integrated to influence current beliefs about hidden states (here, the identity of the hidden jar). The main model was represented as:

\[{b_d}_{ + {\mathrm{1}}}\; = \;{\omega _{\mathrm{1}}} \bullet {b_d} + {\omega _{\mathrm{2}}} \bullet LLR\]

The *ω*_1_ parameter represented the prior weight reflecting the degree to which evidence presented earlier in the trial contributed to the posterior or updated belief *b_d_*_+1_. The *ω*_2_ parameter represented the likelihood weight reflecting the contribution of new information on posterior beliefs. The models examined via formal model comparison were mostly variants drawing from this expression, with different models incorporating different combinations of these parameters or varying them by bead-ratio condition (for details, Baker et al., 2019). Consistent with this previous work, the winning model included a single *ω*_1_ parameter and an *ω*_2_ parameter for each bead-ratio condition.

An example of a participant who has drawn *n_g_* green beads and *n_b_* blue beads can be used to illustrate how this model was derived. This participant opts to draw a new bead which happens to be blue. We can represent this ratio of the conditional probabilities as:

\[\frac{{P(B|({n_b} + {\mathrm{}}1),\;{n_g})}}{{P(G|({n_b} + {\mathrm{}}1),\;{n_g}){\mathrm{}}}} = \frac{{P(B|{n_b},\;{n_g})}}{{P(G|{n_b},\;{n_g})}}\; \bullet \;\frac{q}{{(1{\mathrm{}} - {\mathrm{}}q)}}\]

The terms *P*(*B*|(*n_b_* + 1), *n_g_*) and *P*(*G*|(*n_b_* + 1), *n_g_*) represent Bayesian posterior beliefs, or the probability of the hidden jar’s identity (*B* or *G*) given the sequence of beads that have been drawn. On the right side of the equation, the terms *P*(*B* | *n_b_, n_g_*) and *P*(*G* | *n_b_, n_g_*) represent the prior beliefs (or posterior beliefs from the last draw), and the *q* and (1–*q*) terms represent Bayesian likelihoods, or the likelihood of drawing a bead consistent or inconsistent with the jar identity, respectively. Note that numerator and denominator would flip if the new bead had been green, and the posterior beliefs would be conditional on (*n_b_*, (*n_g_*+1)). By transforming this equation to log space, this equation can be expressed as:

\[\log \left({\frac{{P(B|({n_b}\, + \;1),\;{n_g})}}{{P(G|({n_b}\, + \;1),\;{n_g}){\mathrm{}}}}} \right)\; = \;log\left({\frac{{P(B|{n_b},\;{n_g})}}{{P(G|{n_b},\;{n_g})}}} \right) + {\mathrm{}}log\left({\frac{q}{{(1\; - \;q)}}} \right){\mathrm{}}\]

Since the \[log\left({{\textstyle{q \over {(1 - q)}}}} \right)\] term corresponds to the log-likelihood ratio (*LLR*), the equation can be simplified to:

\[{b_d}_{ + {\mathrm{1}}}\; = \;{b_d}\; + \;LLR\]

which captures formation of the posterior belief (*b_d_*_+1_) at draw (*d* +1) by adding the *LLR* ( \[log\left({{\textstyle{q \over {(1 - q)}}}} \right)\]), or -*LRR* for a bead inconsistent with the jar identity, to update the prior belief at draw *d* (*b_d_*).

#### Value-Based Decision-Making Model

A parameterized partially observable Markov decision process (POMDP) model was used to dissect the contribution of value-based decision-making to sampling behavior. This model is made up of three hierarchically tiered components: belief updating, expected value comparison, and choice.

For the first, fitted probability estimates (e.g., *Π(B)*) were drawn from the winning belief-updating model described above in lieu of the objective Bayesian posteriors. This induces path-dependence in expected values, as discussed in Ashinoff et al. (2021).

For the second, expected values for the choice to guess the jar identity (e.g., the blue jar) or to draw another bead were respectively given by:

\[\begin{array}{l}
{Q_B}\left({{n_b},{n_g}} \right) = {C_{endowment}} + C{'_{draw}} \cdot \left({{n_b} + {n_g}} \right) + {C_{error}} \cdot \Pi (G)\\
\,\,\,\,\,\,\,\,\,\,\,\,\,{Q_{draw}}\, = \,\Pi (G) \cdot \left[ {V\left({{n_b},{n_{g + 1}}} \right) \cdot P\left({g{\mathrm{|}}G} \right) + V\left({{n_{b + 1}},{n_g}} \right) \cdot P\left({b{\mathrm{|}}G} \right)} \right]\\
\,\,\,\,\,\,\,\,\,\,\,\,\,\,\,\,\,\,\,\,\,\,\,\,\,\, + \Pi (B) \cdot \left[ {V\left({{n_b},{n_{g + 1}}} \right) \cdot P\left({g{\mathrm{|}}B} \right) + V\left({{n_{b + 1}},{n_g}} \right) \cdot P\left({b{\mathrm{|}}B} \right)} \right]
\end{array}\]

In the above, *C_endowment_* and *C_error_* were fixed as their true values drawn from the task (*C_endowment_* = 30, *C_error_* = –$15). In contrast, \[C{'_{draw}}\] was itself calculated via the expression \[C{'_{draw}} = - \$ 0.30 + {C_{sub}}\], where *C_sub_* was a fitted parameter allowed to vary by bead-ratio condition reflecting the added relative subjective cost of drawing a bead (beyond the objective cost of –$0.30). Note that *C_sub_* can also be interpreted as the subjective cost of an error. Because the *C_sub_* parameter can only be interpreted in relative terms (i.e., the subjective cost of drawing a bead *relative* to the cost of an error), it is important to acknowledge that it is not possible to differentiate between a reduction in subjective sampling costs and an increase in subjective error costs. Post-hoc sensitivity analyses evaluated a model with a single *C_sub_* parameter shared across bead-ratio conditions.

Finally, choice behavior was implemented via a softmax rule, with an inverse temperature *γ* capturing choice stochasticity. This meant, for example, that the probability of guessing the blue jar was calculated using:

\[{\gamma _{_B}}\left({{n_b},{n_g}} \right) = \frac{{{e^{{Q_B}\left({{n_b},{n_g}} \right) \cdot \gamma }}}}{{({e^{{Q_B}\left({{n_b},{n_g}} \right) \cdot \gamma }}) + ({e^{{Q_G}\left({{n_b},{n_g}} \right) \cdot \gamma }}) + ({e^{{Q_{draw}}\left({{n_b},{n_g}} \right) \cdot \gamma }})}}\]

#### Model Fitting and Comparison

Both the belief-updating and the parameterized POMDP models were fit using a similar process. Fitting was carried out separately for each model and for each participant. This involved minimizing the root mean squared error (RMSE) between estimated and actual probabilities in the belief-updating models and maximizing the mean likelihood estimate (MLE) for actual choices in the POMDP variants. Formal model comparison procedures accounted for fit and complexity by calculating Bayesian Information Criteria (BIC), again for each model and participant. These procedures included the testing of 10 belief-updating model variants and one parameterized POMDP variant, consistent with previous research (Baker et al., 2019).

#### Accounting for Heterogeneity

Because our sample was comprised of participants that belonged to preexisting groups characterized by factors to which one cannot be randomly assigned (e.g., sex, race/ethnicity), statistically controlling for these variables would be an invalid approach (Miller & Chapman, 2001; Verona & Miller, 2015). Thus, we instead included each variable that could be meaningful as an independent variable in analyses, which is a standard recommendation for addressing such potential confounds (Miller & Chapman, 2001; Verona & Miller, 2015). Ultimately, only results with income included in analyses were reported given conceptual and statistical reasons, including previous research that has demonstrated a contribution of income to sampling behavior among psychiatric patients (Baker et al., 2019).

## Results

### Sample Characteristics

Four participants belonging to the clinical diagnostic group were excluded from analyses. The reasons for these four exclusions were: (1) not demonstrating a main effect of bead-ratio condition and drawing more beads in the 100:0 bead-ratio condition than in the 60:40 bead-ratio condition, (2) demonstrating accuracy less than 62%, (3) having an outlier *ω_1_* parameter estimate, and (4) having outlier *C_sub_* parameter estimates in all bead-ratio conditions. Results were unchanged when stricter exclusion criteria were applied (i.e., exclusion of any participant who selected more than one bead in the 100:0 bead-ratio condition). Following exclusions, the sample (*n* = 58) was comprised of 35 individuals with a psychiatric diagnosis (60.3%) and 23 healthy controls (39.6%). Primary psychiatric diagnoses of individuals in the clinical group were OCD (*n* = 18) and GAD, SAD, or panic disorder (*n* = 17). The sample included 25 male and 32 female participants with an average age of 27.67 (*SD* = 7.32). Participants identified as White (*n* = 34; 58.6%), Asian/Asian American (*n* = 10; 17.2%), Black/African American (*n* = 7; 12.1%), mixed race (*n* = 2; 3.4%), and other (*n* = 2; 3.4%). Participants either held full-time employment (*n* = 17; 29.8%), were students (*n* = 19; 33.3%), or were employed part-time or unemployed (*n* = 21; 36.2%). Most participants held a bachelor’s degree or higher (*n* = 41; 71.9%) and reported an annual income of less than $10,000 per year (*n* = 44; 75.9%). The clinical and non-clinical groups demonstrated comparable annual income levels, *χ^2^*(2) = 0.04, *p* = 0.98, *d* = 0.05 (*95% CI* = –0.81 – 0.76), and years of education, *t*(55) = 0.99, *p* = 0.32, *d* = 0.26 (*95% CI* = –0.26 – 0.81). One participant declined to provide demographic information.

Participants with a clinical diagnosis endorsed more trait anxiety (*M(SD)* = 55.91(10.47)), *t*(55) = –10.65, *p <* 0.001, *d* = –2.87 (*95% CI* = –3.62 – –2.12), and state anxiety (*M(SD)* = 46.03(12.89)), *t*(55) = –5.82, *p* < 0.001, *d* = –1.57 (*95% CI* = –2.17 – –0.96), relative to participants without a clinical diagnosis (*M(SD)* = 29.52(6.76), *M(SD)* = 28.39(8.06), respectively). There were no differences by annual income level in trait anxiety, *F*(2,53) = 0.33, *p* = 0.72, *η_p_^2^* = 0.01 (*95% CI* = 0 – 0.02), or state anxiety, *F*(2 53) = 0.04, *p* = 0.96, *η_p_^2^* = 0.002 (*95% CI* = 0 – 0.02).

### Task Behavior

#### Accuracy and Probability Estimates

**Figure 1:**
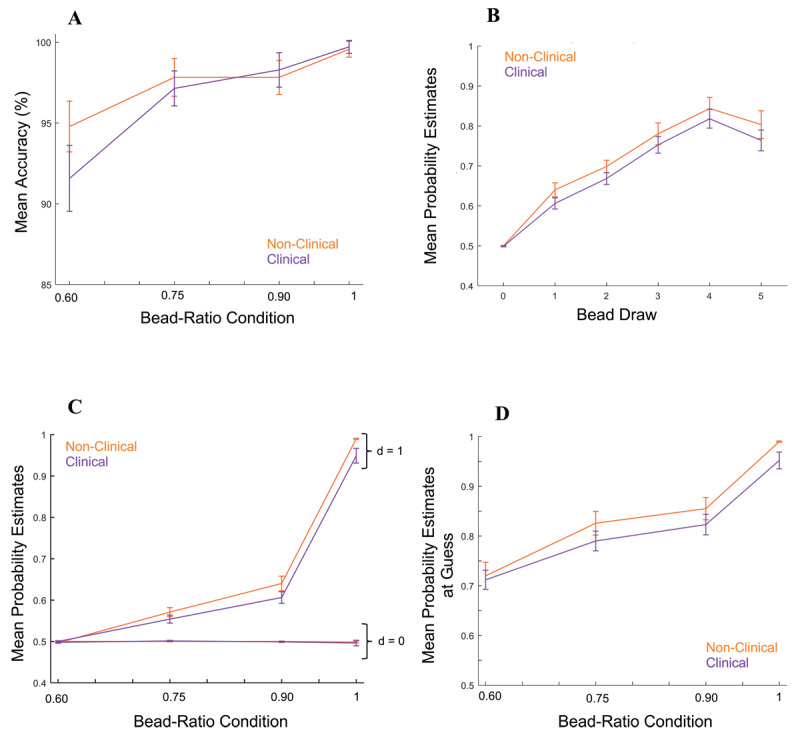
*Task Behavior for Clinical and Non-Clinical Groups*. **(A)** Accuracy by clinical group for each bead-ratio condition. **(B)** Mean probability estimates before each bead draw by clinical group across correct trials in the 90:10 bead-ratio condition. **(C)** Mean probability estimates for before (draw 0; d = 0) and after (draw 1; d = 1) the first bead draw by clinical group across bead-ratio conditions. **(D)** Mean probability estimates at guess (i.e., probability estimate provided immediately before guessing the identity of the jar) by clinical group across bead-ratio conditions.

Repeated-measures ANOVAs were used to test for differences in task performance between diagnostic groups. Non-significant interactions between clinical group and bead-ratio condition indicated that there were no clinical group differences across bead-ratio conditions in accuracy, *F*(1,56) = 0.63, *p* = 0.42, *η_p_^2^* = 0.01 (*95% CI* = 0 – 0.12) (Figure 1A), or mean probability estimates, *F*(1,56) = 1.34, *p* = 0.25, *η_p_^2^* = 0.02 (*95% CI* = 0 – 0.14) (Figure 1B shows mean probability estimates by bead draw in one bead-ratio condition). A non-significant interaction between clinical group and bead-ratio indicated that the clinical and non-clinical groups also demonstrated comparable mean probability estimates across bead-ratio conditions before (draw 0), *F*(1,56) = 0.39, *p* = 0.53, *η_p_^2^* = 0.007 (*95% CI* = 0 – 0.10), and after the first bead draw (draw 1), *F*(1,56) = 0.72, *p* = 0.39, *η_p_^2^* = 0.01 (*95% CI* = 0 – 0.12) (Figure 1C), and at guess, *F*(1,56) = 1.88, *p* = 0.77, *η_p_^2^* = 0.03 (*95% CI* = 0 – 0.16) (Figure 1D). Taken together, results suggest that participants engaged in the task as intended and that there were no clear differences in probability estimates between the clinical and non-clinical groups.

#### Draws-to-Decision

**Figure 2:**
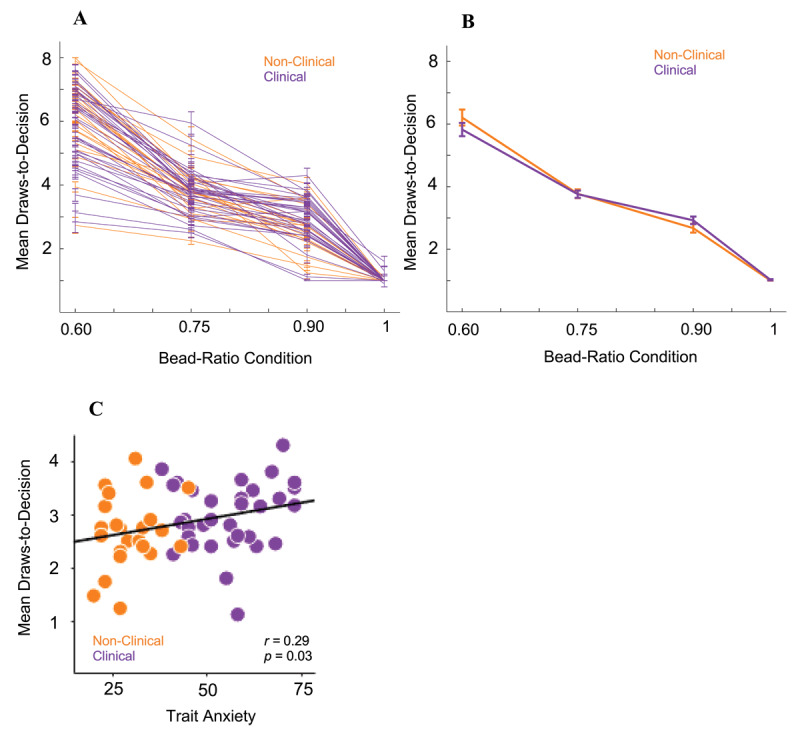
*Draws-to-Decision Behavior for Clinical and Non-Clinical Groups*. **(A)** Mean draws-to-decision across bead-ratio conditions for individual participants and **(B)** averaged by clinical group. **(C)** Correlation of mean draws-to-decision in the 90:10 bead-ratio condition and trait anxiety.

Figure 2 shows mean draws-to-decision across bead-ratio conditions for all participants (Figure 2A provides individual trajectories; Figure 2B shows group averages). A repeated-measures ANOVA revealed a significant interaction of bead-ratio condition by clinical group when predicting mean draws-to-decision across bead-ratio conditions, *F*(2,114) = 4.22, *p* = 0.02, *η_p_^2^* = 0.06 (*95% CI* = 0.002 – 0.16). However, post-hoc tests revealed no differences between the clinical and non-clinical groups in draws-to-decision for any individual bead-ratio condition, all *p*-values > 0.2. A repeated-measures ANOVA demonstrated a significant interaction of continuously measured trait anxiety and bead-ratio condition when predicting draws-to-decision, *F*(2,114) = 3.17, *p* = 0.04, *η_p_^2^* = 0.05 (*95% CI* = 0.001 – 0.14), suggesting a transdiagnostic effect of trait anxiety on sampling behavior across bead-ratio conditions. Post-hoc analyses revealed that the correlation of trait anxiety and mean draws-to-decision was most robust in the 90:10 bead-ratio condition, *r* = 0.29 (*95% CI* = 0.03 – 0.51), *p* = 0.03 (Figure 2C). As such, subsequent analyses focused on the 90:10 bead-ratio condition. There were no differences in mean draws-to-decision in any bead-ratio condition by annual income level, all *p-*values > 0.30.

### Explanatory Computational Models of Increased Information Sampling

**Figure 3:**
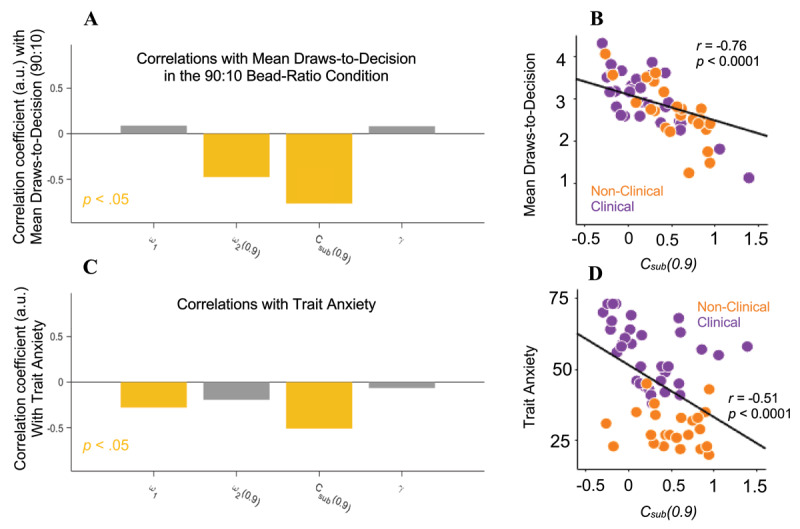
*Model-Based Analyses*. **(A)** Correlation of mean draws-to-decision in the 90:10 bead-ratio condition with key computational parameters from potential explanatory models and **(B)** *C_sub_(0.9)* specifically. **(C)** Correlation of trait anxiety with key computational parameters from potential explanatory models and **(D)** *C_sub_(0.9)* specifically.

We next sought to clarify the mechanisms underlying the association between trait anxiety and draws-to-decision in the 90:10 bead-ratio condition. Two potential explanatory models were tested given previous research which has suggested that increased information sampling in clinical patients may arise from alterations in inferential processes (Baker et al., 2019) or value-based sampling decisions (Hauser et al., 2017). As in previous work, we first modeled inferential processes associated with draw-by-draw probability estimates in the task using a belief-updating model and then augmented this inferential model within a POMDP framework to explain sampling (drawing versus guessing) decisions. Correlations between key computational parameters and behavioral outcomes are summarized in Figure 3A–D. Interparameter correlations are reported in Table 2A–C.

**Table 2:** *Interparameter Correlations*. Interparameter correlations for **(A)** belief-updating model, **(B)** value-based decision-making model, and **(C)** between models.


A	*ω*_2_ *(0.6)*		*ω*_2_ *(0.75)*		*ω*_2_ *(0.90)*	
		
	*r (95%CI)*	*p*	*r (95%CI)*	*p*	*r (95%CI)*	*p*

*ω* ***1***	–0.15	0.24	–0.05	0.70	0.01	0.93

	(–0.39 – 0.11)		(–0.30 – 0.21)		(–0.25 – 0.27)	

**B**	***C_sub_(0.6)***		***C_sub_(0.75)***		***C_sub_(0.9)***	
		
	***r (95%CI)***	***p***	***r (95%CI)***	***p***	***r (95%CI)***	***p***

*γ*	0.18	0.16	0.17	0.19	0.20	0.14

	(–0.08 – 0.42)		(–0.09 – 0.41)		(–0.06 – 0.44)	

**C**	***C_sub_(0.6)***		***C_sub_(0.75)***		***C_sub_(0.9)***	
		
	***r (95%CI)***	***p***	***r (95%CI)***	***p***	***r (95%CI)***	***p***

*ω* ***1***	0.15	0.23	0.48	0.001	0.16	0.21

	(–0.10 – 0.40)		(0.25 – 0.67)		(–0.09 – 0.41)	

*ω* **_2_** ***(0.6)***	0.13	0.31				

	(–0.12 – 0.38)					

*ω* **_2_** ***(0.75)***			0.35	0.008		

			(0.09 – 0.55)			

*ω* **_2_** ***(0.90)***					0.39	0.002

					(0.14 – 0.58)	


**Figure 4:**
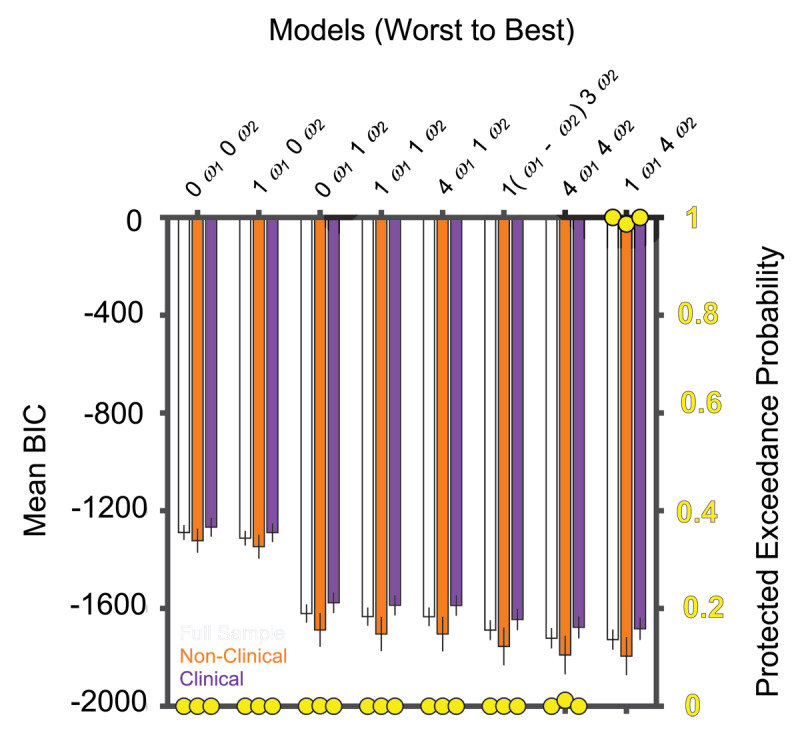
*Mean Bayesian Information Criteria (BIC) and Protected Exceedance Probability for Variants of the Belief-Updating Model*.

First, we identified a belief-updating model that best captured reported probability estimates using the previously described model fitting and comparison procedures (Figure 4). The winning model included a *ω*_1_ parameter representing the prior weight and three *ω*_2_ parameters, one for each bead-ratio condition, representing the likelihood weight. To test if alterations in belief-updating accounted for the association between greater trait anxiety and increased information sampling in the 90:10 bead-ratio condition, we assessed whether relevant belief-updating parameters (i.e., *ω_1_, ω*_2_*(0.9)*) corresponded with both draws-to-decision in the 90:10 bead-ratio condition and trait anxiety. Although lower *ω_1_* was associated with higher trait anxiety, *r* = –0.28 (*95% CI* = –0.50 – –0.02), *p* = 0.03, suggesting that individuals with greater trait anxiety exhibited more prior underweighting, *ω_1_* was not associated with draws-to-decision in the 90:10 bead-ratio condition. Conversely, *ω*_2_*(0.9)* demonstrated a correlation with draws-to-decision in the 90:10 bead-ratio condition, *r* = –0.47 (*95% CI* = –0.65 – –0.24), *p <* 0.001, but no association with trait anxiety.

Given that neither *ω_1_* nor *ω*_2_*(0.9)* adequately explained the association of trait anxiety and greater information sampling in the 90:10 bead-ratio condition, we next examined relevant parameters from the parameterized POMDP model (i.e., *C_sub_(0.9), γ*) that utilized fitted probability estimates from the belief-updating model. Lower relative subjective cost in the 90:10 condition *C_sub_(0.9)* was associated with more draws-to-decision in this condition, *r* = –0.75 (*95% CI* = –0.84 – –0.61), *p <* 0.0001, and with more trait anxiety, *r* = –0.48 (*95% CI* = –0.66 – –0.25), *p <* 0.0001, suggesting that individuals who perceived the relative cost of drawing versus incorrectly guessing to be smaller had greater trait anxiety and engaged in greater information sampling. There was no association between choice stochasticity *γ* and either draws-to-decision in the 90:10 bead-ratio condition or trait anxiety.

Although the effect of trait anxiety on draws-to-decision behavior was most robust in the 90:10 bead-ratio condition, there was evidence for anxiety-related alterations in valuation across bead-ratio conditions, which was reflected in draws-to-decision behavior. Specifically, *C_sub_(0.6), C_sub_(0.75)*, and *C_sub_(0.9)* were all correlated with trait anxiety and mean draws-to-decision in the respective condition (Table 1).

**Table 1:** *Correlations*. Correlation of *C_sub_* parameters for each bead-ratio condition and the shared *C_sub_* parameter across bead-ratio conditions with trait anxiety and mean draws-to-decision in the respective bead-ratio condition, as well as partial correlations (“adjusted”) accounting for shared variance between *C_sub_* and inverse temperature *γ* parameters.


	*C_sub_(0.6)*		*C_sub_(0.75)*		*C_sub_(0.9)*		*shared C_sub_*	
			
	*r*	*p*	*r*	*p*	*r*	*p*	*r*	*p*

**Trait Anxiety**	–0.21	0.054	–0.35	0.004	–0.48	<0.001	–0.12	0.28

**Trait Anxiety, adjusted**	–0.23	0.047	–0.36	0.003	–0.48	<0.001	–0.35	0.004

**Mean DTD(0.6)**	–0.56	<0.001					–0.54	<0.001

**Mean DTD(0.6), adjusted**	–0.65	<0.001					–0.54	<0.001

**Mean DTD(0.75)**			–0.28	0.02			–0.43	<0.001

**Mean DTD(0.6), adjusted**			–0.39	0.002			–0.47	<0.001

**Mean DTD(0.9)**					–0.75	<0.001	–0.26	0.02

**Mean DTD(0.9), adjusted**					–0.76	<0.001	–0.56	<0.001


**Note:** DTD = draws-to-decision.

### Post-Hoc and Sensitivity Analyses

Analyses primarily focused on the 90:10 bead-ratio condition because this was the bead-ratio condition in which the effect of trait anxiety on sampling behavior was most robust. However, the effect of trait anxiety on sampling behavior was observed across all bead-ratio conditions and did not appear to be specific to the 90:10 bead-ratio condition. Thus, condition-general valuation alterations were examined using an alternative model that included one shared subjective cost parameter for all bead-ratio conditions. This model demonstrated poorer fit than the model with *C_sub_* parameters specified for each bead-ratio condition and produced a shared subjective cost parameter that was highly correlated with the inverse temperature parameter *γ*. In partial correlations adjusting for the shared variance with the inverse temperature parameter *γ*, the shared subjective cost parameter correlated with trait anxiety, *r* = –0.35 (*95% CI* = –0.55 – –0.10), *p* = 0.004, and with mean draws-to-decision in the 90:10 bead-ratio condition, as well as mean draws-to-decision in the other bead-ratio conditions (Table 1). Overall, while this model supported our decision to model subjective cost as a condition-specific parameter, it suggests that a broader alteration in valuation not restricted to a particular condition could plausibly explain the variability in trait anxiety and its observed effects on sampling decisions. Based on the strength of the correlation between the shared *C_sub_* parameter and mean draws-to-decision in the 90:10 bead-ratio condition, it is possible that this general alteration could manifest more strongly in certain conditions.

To assess whether anxiety-related increases in information sampling depended primarily upon inferential or valuation mechanisms, we tested specificity of the observed associations between model parameters (*ω_1_* and *C_sub_(0.9)*) and trait anxiety. In a linear regression model predicting trait anxiety from *ω_1_* and *C_sub_(0.9)*, only *C_sub_(0.9)* retained significance, *t*(54) = –3.27, *p* = 0.002, whereas *ω_1_* did not, *t*(54) = –1.30, *p* = 0.20, suggesting that *C_sub_(0.9)* was indeed driving trait-anxiety associated increased draws-to-decision behavior. A second linear regression was conducted predicting mean draws-to-decision in the 90:10 bead-ratio condition from *ω*_2_*(0.9)* and *C_sub_(0.9)*. Both *ω*_2_*(0.9)* and *C_sub_(0.9)* accounted for significant variance in draws-to-decision, *t*(55) = –2.25, *p* = 0.03, *d* = –0.61, *t*(55) = –5.39, *p* < 0.00001, *d* = –1.45, respectively. Findings reflect expected and dissociable contributions of inference and value-based decision-making on draws-to-decision with effects in the same direction. Overall, our sensitivity analyses strengthen support for the interpretation that while inferential and valuation processes both contribute to draws-to-decision behavior, changes in relative subjective valuation specifically seem to drive trait-anxiety associated effects.

Finally, we examined the specificity of our effects to trait anxiety versus other related clinical constructs. Depression was measured using the Depression, Anxiety, and Stress Scales (DASS; Lovibond & Lovibond, 1996), a 21-item self-report questionnaire with good validity and reliability in other samples (Henry & Crawford, 2005). Although depression was highly related to trait anxiety, *r* = 0.82 (*95% CI* = 0.71 – 0.88), *p <* 0.0001, and demonstrated some association with *C_sub_(0.90), r* = –0.31 (*95% CI* = –0.53 – 0.05), *p* = 0.01, it was not correlated with draws-to-decision in the 90:10 bead-ratio condition, *r* = 0.10 (*95% CI* = –0.34 – 0.16), *p* = 0.43.

## Discussion

Using a well-controlled version of the beads task (Baker et al., 2019), dimensional trait anxiety was found to be correlated with increased information sampling (i.e., more draws-to-decision) in a condition with high objective evidence strength (i.e., 90:10 bead-ratio condition). This was not explained by inferential differences in weighting of new or old information, but rather by different cost-benefit analysis suggesting that individuals with greater trait anxiety placed a higher relative subjective cost to incorrect guesses in comparatively unambiguous contexts.

Previous research has similarly found a correlation between dimensional measures of anxiety and greater information sampling (Crockett et al., 2012) as well as increased information sampling among individuals with psychiatric disorders characterized by high trait anxiety. Indeed, when asked to make a low-risk decision, individuals with OCD requested more information before making a choice (Foa et al., 2003), and individuals high in obsessive-compulsive symptoms requested more repeated information when performing a tone discrimination task (Strauss et al., 2021). This kind of behavior has been proposed to be due in part to overestimation of future threat—that is, the more an individual perceives an anticipated outcome to be negative, the more likely they are to gather additional information to avoid that outcome (Clark & Beck, 2011). Though adequate sensory-perceptual evidence might be available (e.g., the stove dial is in the “off” position), patients with anxiety and compulsive disorders may find that even a very unlikely possibility of this evidence leading to a wrong choice justifies repeatedly sampling information of little incremental value (Sachdev & Malhi, 2005) (e.g., returning to the stove to check if the burner is on). The present study supports this interpretation.

Our finding that valuation of sampling decisions, over and above weighting of information during probabilistic inference, drove trait-anxiety associated sampling behavior in the 90:10 bead-ratio condition is generally consistent with previous research conducted in individuals with anxiety and compulsive disorders. Adolescents with OCD demonstrated increased information gathering before committing to a decision, which was driven by lower subjective sampling costs (Hauser et al., 2017). Other studies have also identified anxiety-related differences in valuation processes, although the tasks used have included conditions where participants sample from unknown distributions. Illustrating this is research showing alterations in value-based computations among individuals with OCD in uncertain task conditions (Pushkarskaya et al., 2015, 2017), as well as associations in general, non-clinical samples between anxiety and compulsivity with the willingness to incur a cost to receive non-instrumental information that would reduce uncertainty but not change future outcomes (Bennett et al., 2021; Charpentier et al., 2022). Introduction of monetary reward incentives has also been shown to minimize differences in speed-accuracy tradeoffs between patients with OCD and healthy controls during perceptual decision-making on a random-dot-motion task under low uncertainty, suggesting a contribution of value-based sampling decisions (Banca et al., 2015).

In contrast to most prior studies, the version of the beads task used in the present study explicitly provided outcome probabilities (i.e., bead-ratios were shown on each trial). It was striking that our findings were most robust in the 90:10 bead-ratio condition, where the evidence was strong and an optimal agent would draw relatively few beads before making a choice because the marginal gain in certainty surrounding the outcome ceases to trade off favorably with the money paid for it. This tendency in high-anxiety individuals toward excessive information sampling, in the condition with the clearest information and without a corresponding deficit in belief-updating, could reflect a specific need to resolve uncertainty surrounding the outcomes of their choices far beyond the optimal strategy. Inferential alterations tend to manifest in weaker evidence conditions, strengthening confidence that valuation alterations explain this behavior. Although sensitivity analyses suggested that a general (not condition-specific) alteration in valuation could explain this anxiety-related phenotype, it is possible this manifested more in the 90:10 bead-ratio condition because other processes (e.g., prior weight *ω_1_*) had a stronger influence on the lower objective evidence strength conditions (as demonstrated in Baker et al., 2019) introducing additional variance masking effects in those conditions.

The present study has several strengths, particularly inclusion of a dimensional measure of anxiety (versus diagnostic group comparisons), a transdiagnostic sample, a modified version of the beads task with enhanced experimental controls, and the use of a computational-modeling approach that enabled us to examine mechanisms. Despite these strengths, the study has several limitations. Like many studies that include patients with a psychiatric diagnosis (Banca et al., 2015; Foa et al., 2003; Hauser et al., 2017; Pushkarskaya et al., 2015, 2017) our clinical group was relatively small, although represents an advantage over most studies examining the association of anxiety and information sampling which have generally only included individuals who have not been diagnosed with a psychiatric disorder. Additionally, although the task included several design features to control for cognitive confounds, not all relevant cognitive factors that could have contributed to task performance (e.g., risk aversion, reward sensitivity) were assessed. This is largely a limitation of the task. In the present study, reward outcomes were deterministic. The task lacked manipulations of reward outcomes (e.g., probabilistic contingencies or outcome variance) that would allow for a more complex valuation model to be evaluated. Future research should consider extending the current task or adding other tasks that specifically probe related cognitive factors, strengthening the ability to establish the specificity of findings. For example, Baker and colleagues (2019) supplemented the incentivized beads task with tasks designed to quantify loss, risk, and ambiguity aversion (Levy et al., 2010; Tom et al., 2007). Although risk and ambiguity aversion were not associated with task performance or clinical measures, loss aversion emerged as a potential explanatory variable of socioeconomic status related influences on draws-to-decision.

Ultimately, understanding the neurocognitive mechanisms of information sampling behavior could lead to novel targets for clinical treatment of anxiety and compulsive disorders. Our results suggest that the processes supporting value-based sampling decisions may be one such potential target. Replication would support developing and testing interventions for anxiety that retrain or recalibrate cost-benefit analysis in valuation processes, meeting the recognized need for mechanistic treatments (Corlett & Schoenbaum, 2021).

## Previously Published Data

A portion of the findings reported in this paper were presented at the 60^th^ Annual Meeting of the American College of Neuropsychopharmacology. *Abstract citation:* Rapp, A. M., Ashinoff, B. K., Baker, S., Simpson, H. B.,* & Horga, G.* (2021). Transdiagnostic anxiety-related increases in information sampling associated with altered valuation. *Neuropsychopharmacology, 46*, 306–307. (*Denotes co-last authors)
